# Supplementary material for: Solid Lipid Nanoparticles Enhancing the Leishmanicidal Activity of Delamanid
Source: Pharmaceutics. 2023 Dec 27;16(1):41. doi: 10.3390/pharmaceutics16010041 (PMC10818933; doi:10.3390/pharmaceutics16010041)
Supplement: Supplementary file 1 [file pharmaceutics-16-00041-s001.zip › pharmaceutics-2731988-supplementary.pdf]

SUPLEMENTARY MATERIAL

Solid lipid nanoparticles enhancing the leishmanicidal activity of Delamanid

Javier Santamaría-Aguirre, Daniela Jacho, Miguel Angel Méndez, Ana Poveda, Javier Carrión, and Mónica L. Fanarraga

Supplementary procedures:

Procedure S1. Molecular Docking

Auto Dock Vina software (http://vina.scripps.edu/index.html) was used for molecular docking with the proteins dihydrofolate reductase – thymidylate synthetase (DHFR-TS) and Topoisomerase (TOP II) from *Leishmania*. Structures, obtained from PubChem, were processed in ChemAxon, Chemicalize tool, and their pka, and pH-dependent distribution curves were calculated. Using the AC/DC and MarvinSketch programs, the charges of the molecules were modified for pH 7; were debugged, optimized, and corrected in Check Structure, and saved in mol and .sdf formats. Based on previous research the human alpha and beta topoisomerase II proteins were selected, their structure was taken from the PDBj and RCSB databases, and their possible amino acids of interest for their binding with ligands, their active centers, and sequences of biological interest were investigated on the Uniprot page. Salmonella gyrase was also selected due to its high affinity for fluoroquinolones and topoisomerase II from *L. amazonensis*. Best binding sites were tested in Docking Server (https://www.dockingserver.com/web). A total of 42 fluoroquinolones and meglumine antimoniate were uploaded as ligands specifying the protonation pH. The proteins to be used were loaded, specifying the chain and the best binding sites previously found. Docking was performed adding partial Gasteiger charges to the ligand; Non-polar hydrogen atoms and rotatable bonds were added. Parent hydrogen atoms, Kollman bonded atom type charges, and solvation parameters were added with the help of AutoDock tools. Affinity maps and 0.375 Å spacing were generated using Autogrid. Distance-dependent dielectric functions and the AutoDock parameter set were used in the calculation of the electrostatic and van der Waals terms. Docking simulations were performed using the Lamarckian genetic algorithm and the local search method, with the initial position, orientation, and torsions of the molecules randomized. All rotational torques were released during coupling. Each docking experiment was derived from two different runs that were set to terminate after a maximum of 250,000 power evaluations for a *n* set to 150. During the search, a translation step of 0.2 Å and quaternion and torsion steps of 5 were applied [34].

Procedure S2. Detection of the DNA TOP II gene in *L. amazonensis*

For detection of the DNA TOP II gene in *L. amazonensis*, a search was carried out for all the gene sequences for this same protein in other species of the genus Leishmania, and the complete genome of our species of interest was obtained. All amino acid sequences for this protein were downloaded from the NCBI Gene platform, and a nucleotide BLAST was performed in MEGA8. The gene sequence was rescued from a similar region corresponding to the total genome of Leishmania, as described by Spíndola et.al [33].

Procedure S3. Fluorescence method for IC50

Promastigotes of *Leishmania* in exponential phase, cultured in Schneider liquid medium with 10% fetal bovine serum (FBS) were used. In a 96-well plate, 100 µl of parasite culture corresponding to a concentration of $1x10^6$ parasites/ml was added. Different concentrations of the drug were then added, performing serial dilutions. Additionally, a control with parasites without drug, a control of substances in Schneider medium for each test concentration, and another control with only Schneider medium supplemented with 10% fetal bovine serum were prepared. The plate was incubated at 25°C. 24 hours later, two washes with PBS were performed; 200 µL of PBS was added, and it was centrifuged for 10 minutes at 4000 rpm. Then, 250 µL of supernatant was removed, and another wash with 200 µL of PBS was performed, followed by centrifugation, and 200 µL of supernatant was removed. The parasites were lysed with 100 µL of lysis buffer to which SYBR Gold 10x (100 µl per 10 ml) was added at the time of plate measurement. The released DNA is proportional to the number of parasites, and the fluorescent intercalator (SYBR Gold 0.1x) intercalates with the DNA and allows reading the plate on a Synergy H1 Biotek fluorescence plate reader; fluorescence is directly proportional to the number of existing parasites. MIC, NIC, and IC50 were determined in the graph obtained by processing the data using the GraphPad Prism 6 program, using the modified Gompertz function [35].

Supplementary figures:

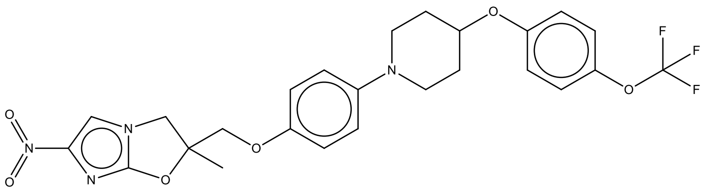

MMV688262 (Delamanid)

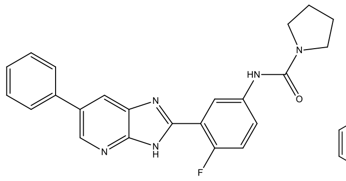

MMV688372

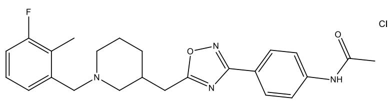

MMV689061

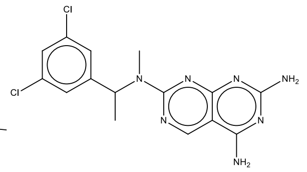

MMV689437

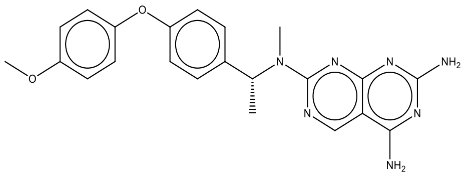

MMV690102

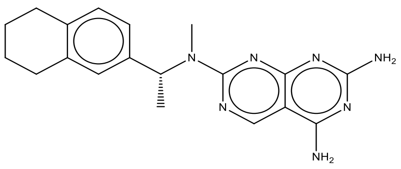

MMV690103

Figure S1. Substances selected from MMV library. Chemical structures of substances selected for their high activity against trypanosomatids and low cytotoxicity against human cells.

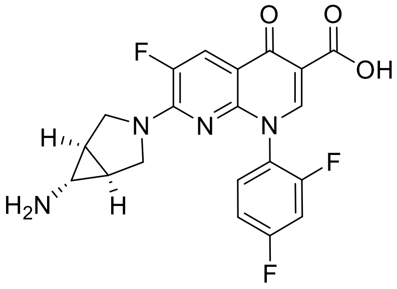

Trovafloxacin

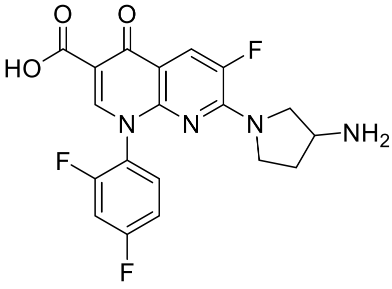

Tosufloxacin

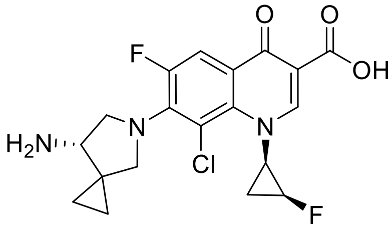

Sitafloxacin

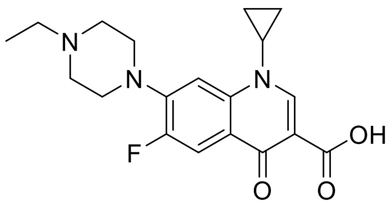

Enrofloxacin

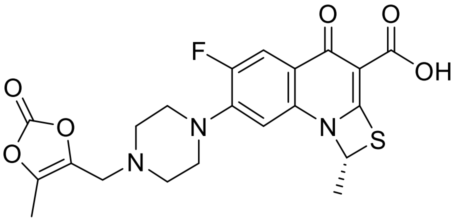

Prulifloxacin

Figure S2. Substances selected through molecular docking, experimental data, and bibliographic review. Chemical structure of the substances with the highest affinity for enzymes of interest according to molecular docking, previous experimental experiences, and scientific articles review.

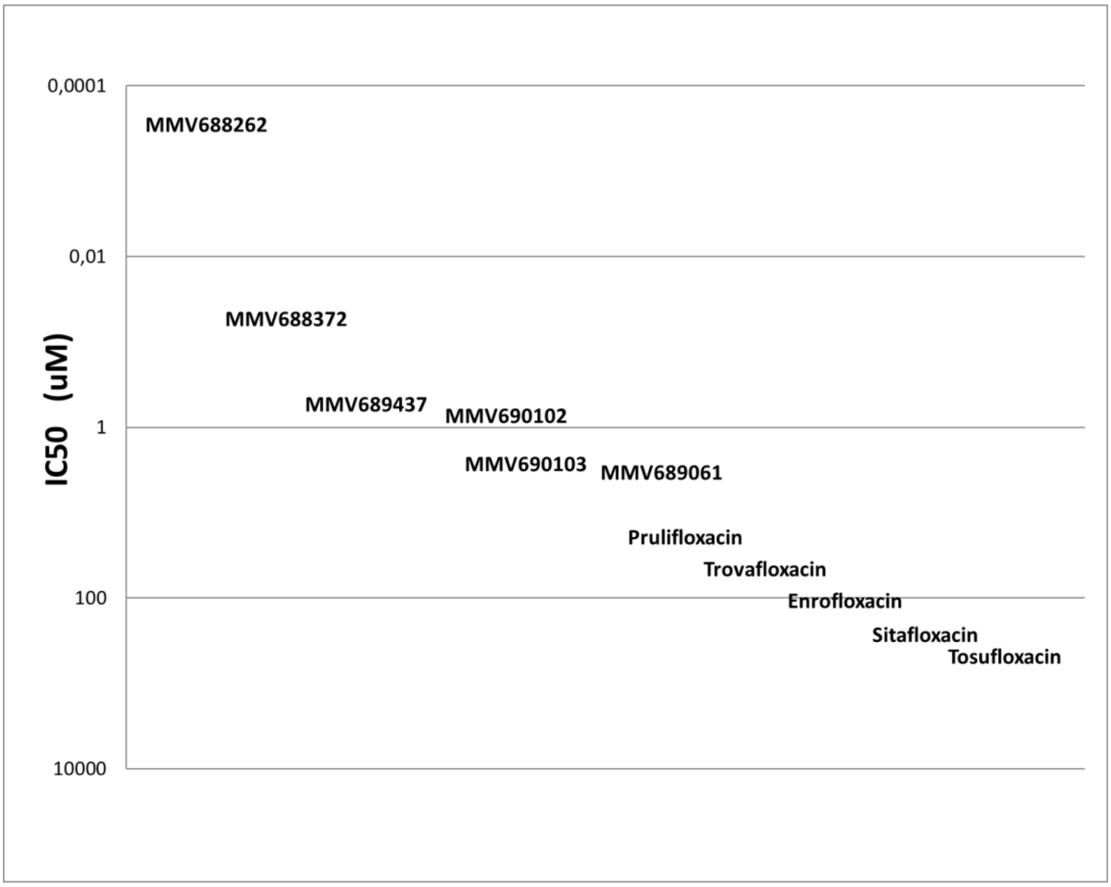

Figure S3. Relative leishmanicidal activity on promastigote. MMV688262 (delamanid) showed activity against *Leishmania* promastigotes at nanomolar concentrations. Prulifloxacin was the fluoroquinolone with the highest activity.

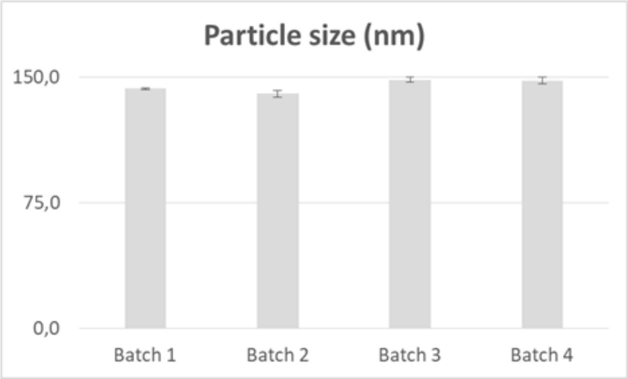

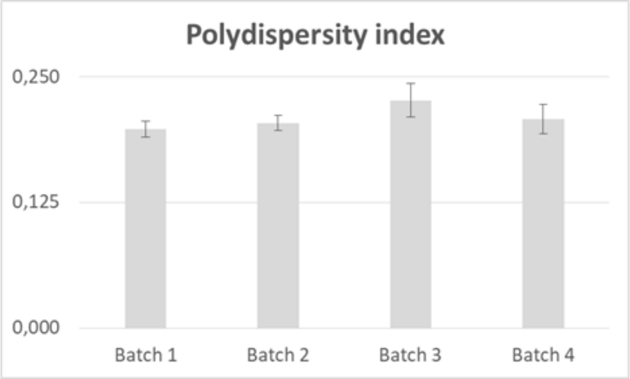

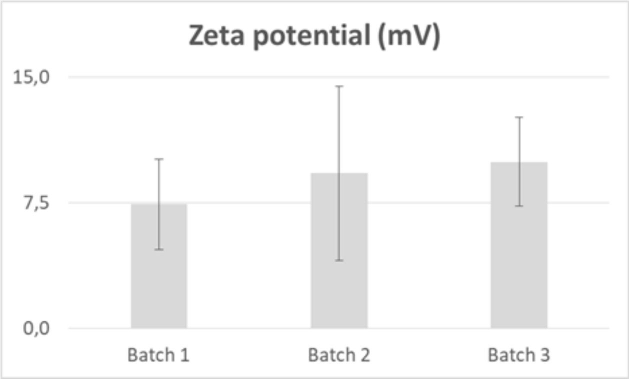

Figure S4. Physical characterization of SLNP batches synthesized independently. The developed manufacturing process generates high reproducibility regarding size, polydispersity index, and zeta potential.

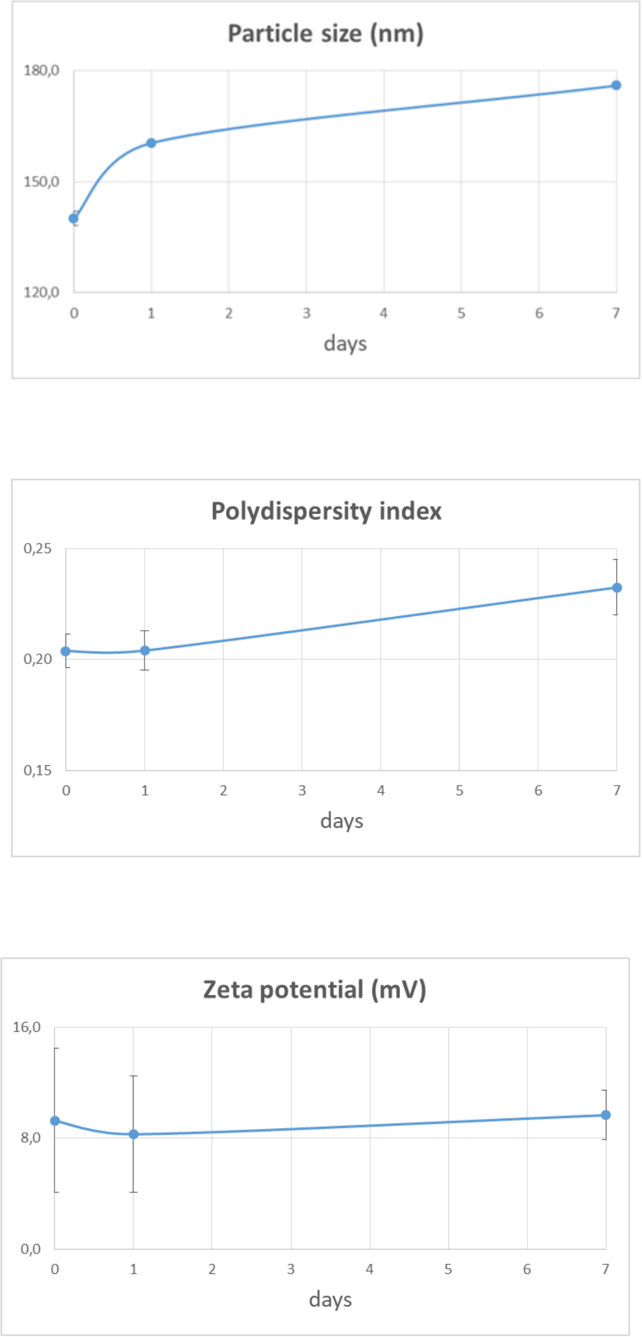

Figure S5. Physical stability of SLNPs. When stored in refrigeration, SLNPs maintain their physical characteristics for at least one week, indicating good physical stability.

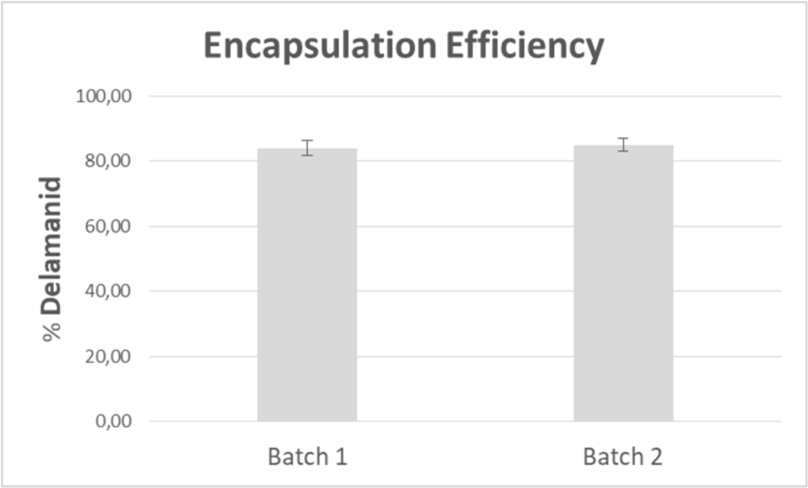

Figure S6. Encapsulation efficiency of SLNPs. The developed synthesis process attains over 80% drug encapsulation efficiency in SLNPs.

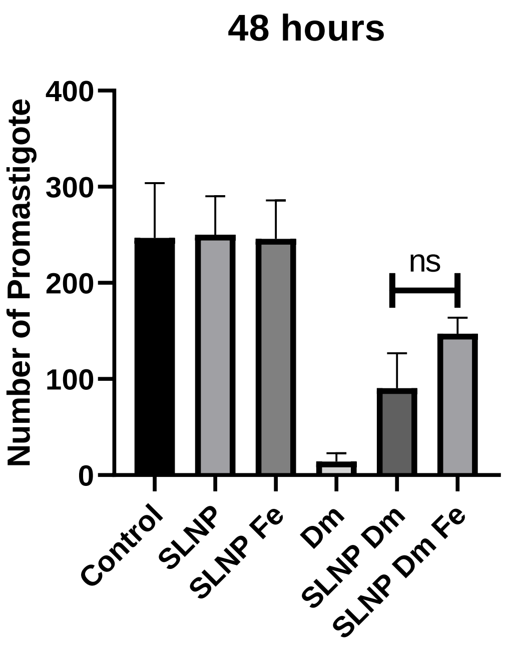

Figure S7. Leishmanicidal activity of SLNPs on promastigotes. The addition of magnetic nanoparticles to SLNPs does not enhance their leishmanicidal activity. In the 48-hour assessment, there were no significant differences in promastigote counts between the untreated control group, the SLNP group (SLNPs without drug), and the SLNP Fe group (SLNPs without drug but with magnetic nanoparticles). delamanid (Dm) in solution displayed the highest activity against promastigotes. Additionally, there were no statistically significant differences observed between SLNP Dm (SLNPs with drug) and SLNP Dm Fe (SLNPs with drug and magnetic nanoparticles).

a

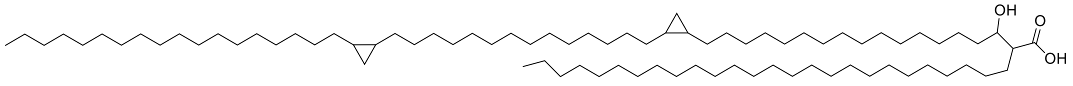

b

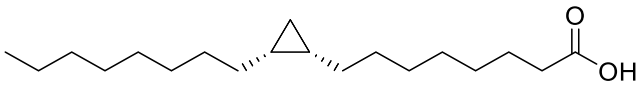

Figure S8. Cyclopropane fatty acids in *Mycobacterium* and *Leishmania*. Structures of the alpha - mycolic acids present in *Mycobacterium* (a), and of a cis-9,10-methyleneoctadecanoic acid (b) present in *Leishmania* and some species of bacteria. In both cases with similar protective functions.
